# Field data-based mathematical modeling by Bode equations and vector fitting algorithm for renewable energy applications

**DOI:** 10.1371/journal.pone.0191478

**Published:** 2018-01-19

**Authors:** A. H. Sabry, W. Z. W. Hasan, M. Z. A. Ab. Kadir, M. A. M. Radzi, S. Shafie

**Affiliations:** 1 Department of Control and Automation, Faculty of Engineering, UPM, Serdang, Malaysia; 2 Department of Electrical and Electronic Engineering, Faculty of Engineering, University Putra Malaysia, Serdang, Malaysia; Universita degli Studi della Tuscia, ITALY

## Abstract

The power system always has several variations in its profile due to random load changes or environmental effects such as device switching effects when generating further transients. Thus, an accurate mathematical model is important because most system parameters vary with time. Curve modeling of power generation is a significant tool for evaluating system performance, monitoring and forecasting. Several numerical techniques compete to fit the curves of empirical data such as wind, solar, and demand power rates. This paper proposes a new modified methodology presented as a parametric technique to determine the system’s modeling equations based on the Bode plot equations and the vector fitting (VF) algorithm by fitting the experimental data points. The modification is derived from the familiar VF algorithm as a robust numerical method. This development increases the application range of the VF algorithm for modeling not only in the frequency domain but also for all power curves. Four case studies are addressed and compared with several common methods. From the minimal RMSE, the results show clear improvements in data fitting over other methods. The most powerful features of this method is the ability to model irregular or randomly shaped data and to be applied to any algorithms that estimating models using frequency-domain data to provide state-space or transfer function for the model.

## Introduction

In power systems, the load profile or the power curve is a trend that demonstrates the deviation in demand or electrical load over a definite time. The generation companies apply this information to map how much power they will need to produce at any time. Wind and solar power output fluctuate immensely and are only moderately controllable. The rate of power can be forecasted with a particular accuracy. System engineers build systems to harness any solar and wind resources accessible; therefore, from an operational viewpoint, solar and wind power less resembles the demand load than does the traditional fossil fuel power generation. In some conditions, the wind and solar power resources have been successfully accumulated in connection with electric grids, such as when the wind has a lower rate of penetration and the solar irradiation has a low rate of variation in light intensity. However, the important issue is how can solar and wind power output be extrapolated and the obtained energy be estimated. Therefore, investigating and creating an algorithm to extract a mathematical model is crucial for the interpretation, prediction, and analysis of such power curves. The difficulty is to find an objective criterion and to assign quantitative parameters that govern the trade-off between the smoothness of the power curve and its proximity to the measured data points.

A mathematical model can be defined as an explanation of a system using mathematical theory and language. Modeling may help to describe a system and revise the effects of different elements, in addition to making forecasts about the system’s behavior. In many cases, the value of a scientific field is based on how well the mathematical models created to match the theoretical concepts with the results of repeatable experiments perform.

This research did not cover all the literature regarding this field for the following reasons:

- There is a wide range of fitting, forecasting and modeling techniques that are all based on regression analysis, which is considered a statistical procedure for modeling or estimating the relationships between system parameters and for associating a particular dependent variable with one or more independent variables.
- Many factors affect the shapes of power curves, and there are different factors for different applications, such as wind speed perturbations for wind energy, light shading variations for a solar power rate curve, and parameters that quantitatively describe the load power consumption shape of residential applications such as the switching effects and the power supply structure of household appliances.
- This research proposes a general modeling technique to extract formulas that follow the behavior of the system that is expressed by its observation data points. This technique has the potential to fit and formulate most shapes of curves based only on field or experimental data measurements.

A general outline for the development of this modeling solution is presented, followed by an elaboration of the detailed steps. In Section 2, we introduce the current modeling equations and a list of mathematical data fitting models along with the position of the proposed model among the power curve models in the literature. We first address the previous related work on three trends according to their application within green energy areas: wind, solar and load power profiles in a residential application. Then, the VF algorithm is described and reviewed as the method to be modified and on which this work is based. In Section 3, we provide a survey of vector fitting, while in Section 4, the methodology of the research used to develop the BEVF algorithm, depicted by a diagram to demonstrate the concept, is presented. The proposed algorithm is divided into three phases, Input Adaptor, VF Process, and Output Adaptor, to produce the proposed system equations. In Section 4, the paper presents the models for four features of the power profiles in green energy applications: two in the wind energy application area, one in the solar power field, and one in the modeling of the load demand power curve. These cases were selected based on the availability of their data and previous studies. The results of the proposed technique and a comparative evaluation is also presented. Finally, Section 5 provides concluding remarks as well as future studies.

### The current modeling equations

Interpolation and the least squares method are two commonly used techniques for curve fitting and modeling. Interpolation has normal polynomials and spline representations, while the least squares curve fitting also uses a simple polynomial and is a general approach that permits additional choices of least squares fitting functions such as Chebyshev or spline series. Other fitting methods in the literature include the spline function, which is a curve built from polynomial segments that are subjected to the conditions at their joints. [1] presented an algorithm that includes the cubic smoothing spline. The common model names and their associated equations can be classified as shown in Table 1.

**Table 1:** A List of some mathematical data fit model equations.

Model Names	Degree	Equations
**Polynomial Model**	1 2 3 8	*Y* = *p*_1_*x* + *p*_2_ *Y* = *p*_1_*x*^2^ + *p*_2_*x* + *p*_3_ *Y* = *p*_1_*x*^3^ + *p*_2_*x*^2^ + … + *p*_4_ *Y* = *p*_1_*x*^9^ + *p*_2_*x*^8^ + … + *p*_10_
**Exponential Model**	1 2	*Y* = *ae*^*bx*^ *Y* = *ae*^*bx*^ + *ce*^*dx*^
**Fourier Series Model**	1 2 3 .8	*Y* = *a*_*o*_ + *a*_1_*cos*(*xp*) + *b*_1_*sin*(*xp*) *Y* = *a*_*o*_ + *a*_1_*cos*(*xp*) + *b*_1_*sin*(*xp*)…+*a*_2_*cos*(2*xp*) + *b*_2_*sin*(2*xp*) *Y* = *a*_*o*_ + *a*_1_*cos*(*xp*) + *b*_1_*sin*(*xp*)…+*a*_3_*cos*(3*xp*) + *b*_3_*sin*(3*xp*) *Y* = *a*_*o*_ + *a*_1_*cos*(*xp*) + *b*_1_*sin*(*xp*)…+*a*_8_*cos*(8*xp*) + *b*_8_*sin*(8*xp*)
**Gaussian Model**	1 2 3 4	$Y=a_{1}e^{{-(\frac{x-b_{1}}{c_{1}})}^{2}}$ $Y=a_{1}e^{{-(\frac{x-b_{1}}{c_{1}})}^{2}}+a_{2}e^{{-(\frac{x-b_{2}}{c_{2}})}^{2}}$ $Y=a_{1}e^{{-(\frac{x-b_{1}}{c_{1}})}^{2}}+\ldots +a_{3}e^{{-(\frac{x-b_{3}}{c_{3}})}^{2}}$ $Y=a_{1}e^{{-(\frac{x-b_{1}}{c_{1}})}^{2}}+\ldots +a_{8}e^{{-(\frac{x-b_{8}}{c_{8}})}^{2}}$
**Power Model**	1 2	*Y* = *ax*^*b*^ *Y* = *ax*^*b*^ + *c*
**Rational Model**	02 12 55	$Y=\frac{p_{1}}{\left(x^{2}+q_{1}x+q_{2}\right)}$ $Y=\frac{\left(p_{1}x^{2}+p_{2}x+p_{3}\right)}{\left(x+q_{1}\right)}$ $Y=\frac{\left(p_{1}x^{5}+\ldots +p_{6}\right)}{\left(x^{5}+\ldots +q_{5}\right)}$
**Sum of Sine Model**	1 2 3 .8	*Y* = *a*_1_*sin*(*b*_1_ * *x* + *c*1) *Y* = *a*_1_*sin*(*b*_1_*x* + *c*_1_) + *a*_2_*sin*(*b*_2_*x* + *c*_2_) *Y* = *a*_1_*sin*(*b*_1_*x* + *c*_1_)+…+*a*_3_*sin*(*b*_3_*x* + *c*_3_) *Y* = *a*_1_*sin*(*b*_1_*x* + *c*_1_)+…+*a*_3_*sin*(*b*_8_*x* + *c*_3_)

**Fig 1:**
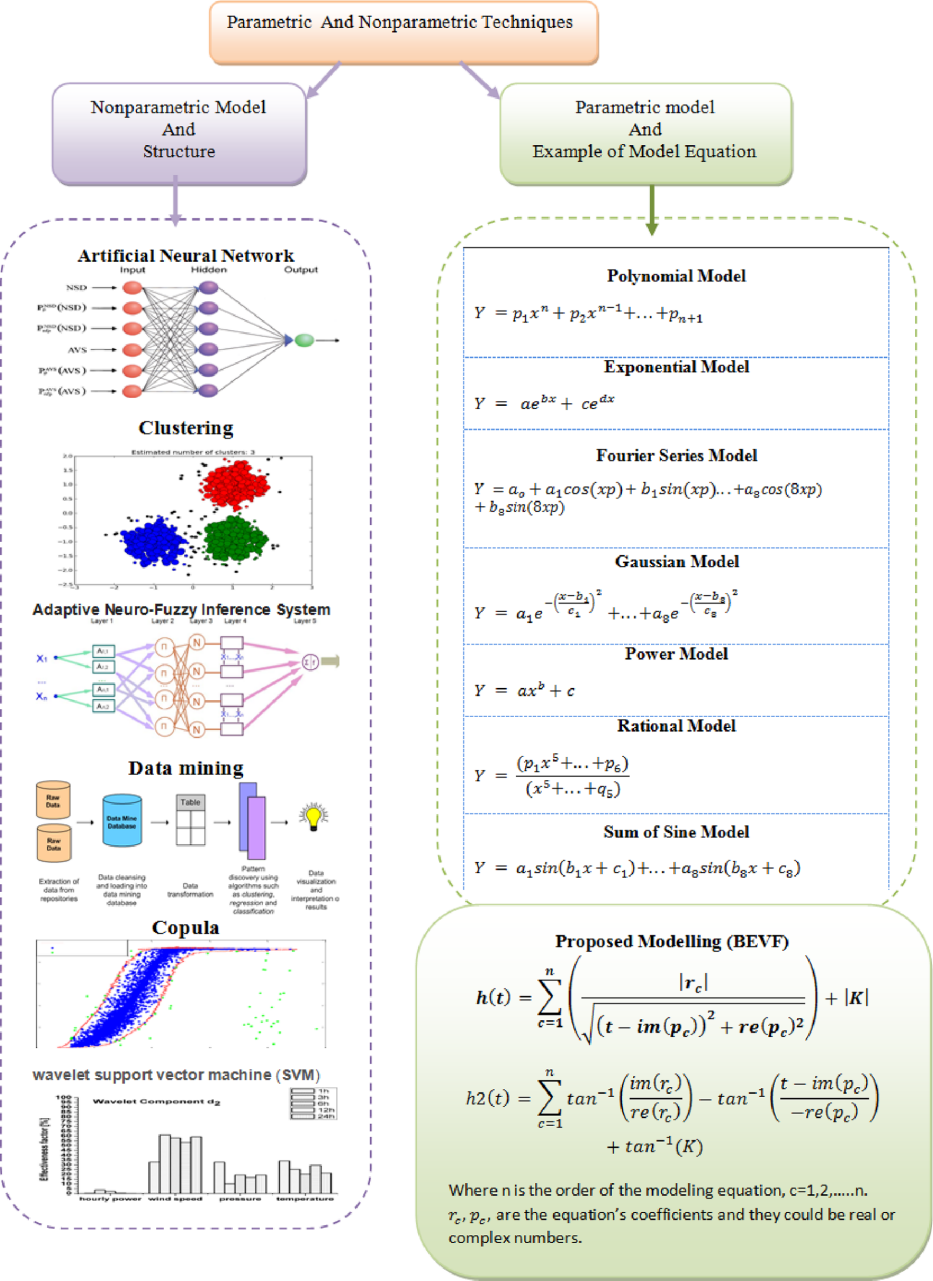
Power curve models and the position of the proposed model.

Table 1 provides a list of the common fitting equations that MATLAB is based on; some of the listed equations may fit one set of data, but not others, and the fitting accuracy depends on the selection of the equation order. There is no dominant fitting approach for the types of observations, even for physical applications. Therefore, it is essential to consider the performance of several statistical methods to fit a particular power curve to select the best one for a given measured data set. A brief classification of most power curve models, with the position of the proposed algorithm included among them, is shown in Fig 1.

### Vector fitting, a survey

Vector fitting (VF) is a robust numerical method for extracting a rational approximation in the frequency domain. The name ‘Vector Fitting’ stems from how the method may be easily generalized to simultaneously fit a vector of system transfer functions [2].

VF is a state equation approximation; thus, the time and the frequency domain approximations are parameterized exactly the same as in the solution of the state matrix. The only difference in the calculation is the basis used during the least squares of the method. The definition of a vector fitting (VF) algorithm is a mathematical method for sampled response-matching system identification, in which the algorithm includes an iterative procedure for solving linear least squares by using partial fraction expansion roots. This technique covers a wide range of variation in frequency domains (from 0-GHz), avoiding ill-conditioned computations and therefore working in a more robust and efficient manner. VF is used in modeling of different electrical systems [3] and other areas such as in filter design [4, 5], in power network analysis [4, 6] and in the field of electromagnetic simulation [7].

VF was an idea first introduced for the modeling of power transmission lines as transient response-measured data in [8]. The fundamental idea of VF is to update the proposed or initialized poles with estimated values as an improved set of poles through the technique of pole relocation, improving the approximation iteratively. VF has the capability to estimate an original system as a new model by means of partial fraction roots of real, complex conjugate, or mixed poles. For better integration and performance of VF, several simplifications and expansions have been considered with special classification [9–17].

## Proposed mathematical modeling algorithm

**Fig 2:**
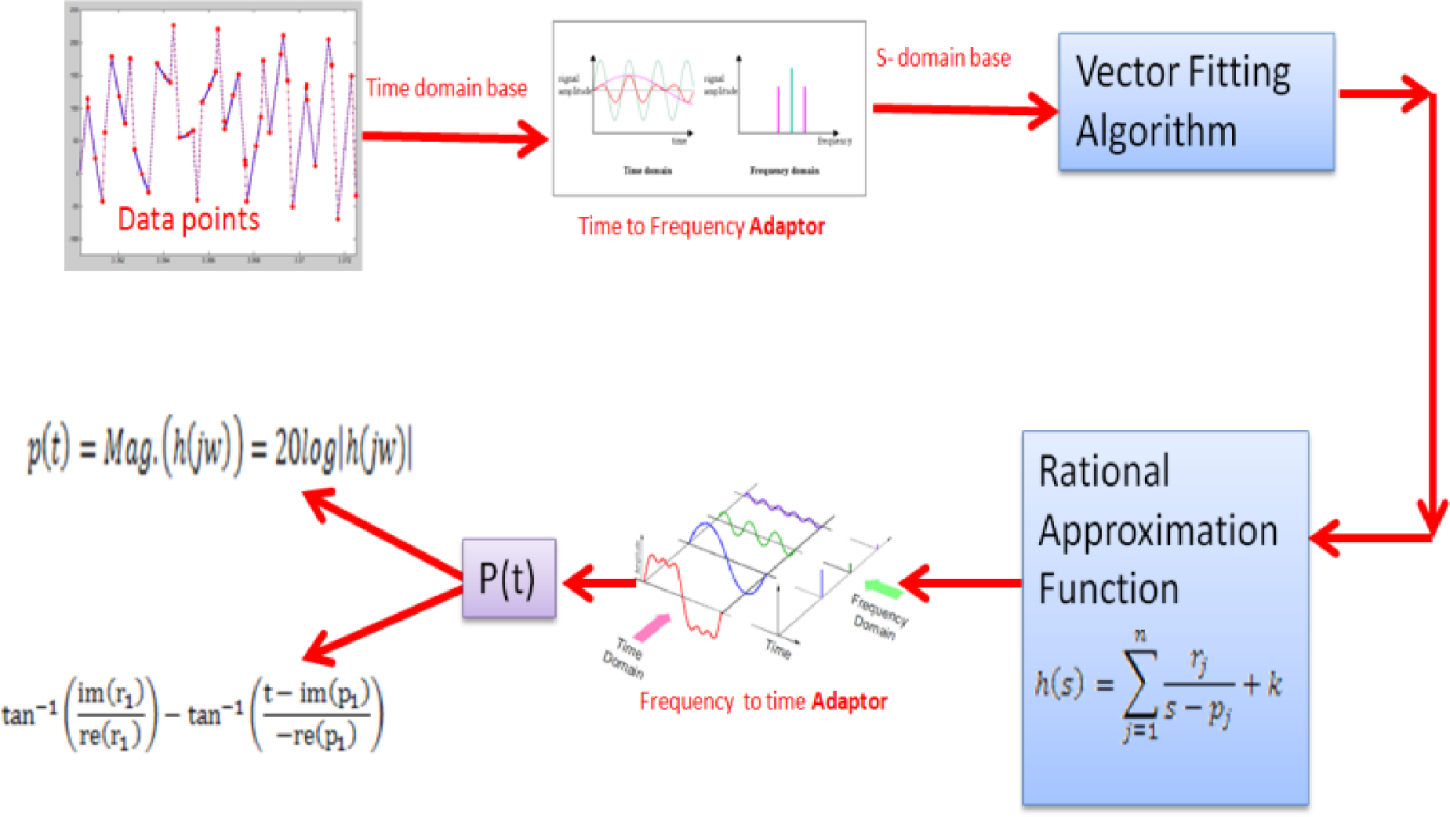
Block diagram of the proposed algorithm.

The Bode equation vector fitting (BEVF) method is a modified algorithm in which the vector fitting (VF) algorithm has been adopted due to its robust numerical fitting property. However, VF provides a state space modeling representation for measured input data in the frequency domain; therefore, the proposed effort investigates the ability of this method to develop a new mathematical model by relying on mathematical assumptions, set as adaptors, to match any measured or calculated data points using the VF process. Thus, the proposed algorithm can be divided into three phases, Input Adaptor, VF Process, and the Output Adaptor, which produce the proposed system’s mathematical equations (Fig 2).

### Input adaptor

This stage represents the assumptions for synthesizing the input data to frequency-domain based algorithm (VF algorithm in this case) and to initialize the parameters required for the second stage. Appendix A shows the software steps with example of empirical data.

Fig 2 depicts the proposed algorithm in which the input data or observations must first be expressed in terms of Cartesian coordinates *f*(*x*)_*k*_ + *j*_0_ = *r*_*k*_, with zero imaginary part (*j*0) or can be represented by one of the two selections in polar form expressed in (1).

$$f(x)=Data=\left\{ \begin{array}{c} Cartesian;\;{f(x)}_{k}+j0=r_{k},\;Ɵ=0\\ Polar;\{\begin{array}{l} {f(x)}_{k}={Ɵ}_{k}=r_{k}\\ {f(x)}_{k}={Ɵ}_{k},\;r_{k}=0\;or\;constant \end{array} \end{array}\right.$$ (1)

where the index k = 1, 2, 3… (number of data points).

The data are now ready to be processed by the conventional VF algorithm.

### VF process

VF provides a model system expressed by its state space. Therefore, the VF algorithm can be defined as a method to determine the state space representation of measured or calculated data points [18]. The polynomial expression of the transfer function can be expressed as:

$$f(s)=\frac{a_{0}+a_{1}s+a_{2}s+\ldots +a_{n}s^{n}}{b_{0}+b_{1}s+b_{2}s+\ldots +b_{n}s^{n}}$$ (2)

The rational approximate function of (2) can be expressed as:

$$h(s)=\sum_{j=1}^{n}\frac{r_{j}}{s-p_{j}}+K$$ (3)

where the term k is a constant and is optional, r_j_ represents the residues and p_j_ represents the poles.

Based on (1), which represents the assumption of data, the algorithm transforms the state space model into a Rational Approximation Function (RAF), which can be seen in (3), and then transforms each fraction of (3) into a sum of 1^st^ order fractions. The aim of transforming the fractions into a sum of 1^st^ order fractions is to be able to easily transform them back into either Bode magnitude form or Bode phase form, as explained in Fig 2. Furthermore, the matching process is based on the assumption of the input data being either in Polar or Cartesian form and then being processed as a complex quantity with zero imaginary parts, as described above in (1).

As explained in [18], the VF method first recognizes the poles by solving the problem in the least squares sense of the linear problem:

σ(s)h(s)=f(s) (4)

where

$$\sigma (s)=\sum_{j=1}^{n}\frac{{\overset{ˇ}{r}}_{j}}{s-q_{j}}+1$$ (5)

$$f(s)=\sum_{j=1}^{n}\frac{r_{j}}{s-q_{j}}+K$$ (6)

q_j_ is the initial pole set. (All poles and residues in (5) and (6) are real or complex conjugate pairs, while k is a real number). The assumption that VF adopts is that the poles of h(s), given in (3), should be equal to the zeros that can be calculated as the eigenvalues of a matrix as in (7) [19], page 612.

$$\left\{p_{j}\}=eig(A-B.C^{T}\right)$$ (7)

In (7), A represents a diagonal matrix with the initial poles q_j_, while B is a column of ones vector, and C^T^ is a row vector with the residues $\left\{{\overset{ˇ}{r}}_{j}\right\}$.

The procedure is applied in an iterative mode where (4) to (7) are solved iteratively with the new poles {p_j_} substituting the previous poles {q_j_}. This pole relocation process typically converges in 2–3 iterations. After identifying the poles, the residues of (3) are calculated by solving the equivalent least squares (LS) problem with the new known poles.

Furthermore, the objective of the vector fitting method is to calculate the unknown poles, residues and constant of the rational approximation function equivalent to (3).

$$h(s)=\frac{r_{1}}{s-p_{1}}+\frac{r_{2}}{s-p_{2}}+\ldots ..\ldots +\frac{r_{n}}{s-p_{n}}+K$$ (8)

where r is the residue and p is the pole of the fraction from h(s). n, is assigned by trial and error based on an appropriate RMS error and is therefore selected in a way that provides an accurate fitting and represents the order of the resultant RAF.

Referring to (8), the general numerical expression of *r*_*n*_, and *p*_*n*_, can be expressed in complex form as:

$$r_{n}=im\left(r_{n}\right)j+re\left(r_{n}\right),p_{n}=im\left(p_{n}\right)j+re\left(p_{n}\right)$$ (9)

where im(r_n_) and re(r_n_) are the imaginary and real parts of r_n_, respectively. The same applies for im(p_n_) and re(p_n_).

Applying the inverse Laplace transform on (8) yields h(t) in (10):

$$h(t)=r_{1}e^{p_{1}t}+r_{2}e^{p_{2}t}+\cdots \;\ldots \;\ldots \;\ldots \;\ldots r_{n}e^{p_{n}t}+k\delta_{t}$$ (10)

#### Selection of initial poles

To measure data with resonance peaks, complex conjugates with weak attenuation must be selected as initial poles and the imaginary parts (β) must be set to cover the range of frequencies of interest. Weak attenuations guarantee that the least squares (LS) problem is solved, and the allocation of the pair over the frequency range reduces the probability that the poles must be relocated a long distance, avoiding the need for many iterations.

The pairs of poles should normally be chosen as follows:

$$\alpha_{n}=-\alpha +j\beta ,\alpha_{n+1}=-\alpha -j\beta ,\alpha =\beta /100$$ (11)

Usually, β is assigned to be linearly spaced over the frequency range of interest (recommended choice). In some instances, a logarithmic distribution results in a faster convergence.

#### Output of VF

The fitting process output of the VF algorithm is a state space of the fitted trace, but as a result, it is simple to transform it into the Rational Approximation Function (RAF) form by using a specified MATLAB function.

### Output adaptor

It is worth mentiond that the VF has been addressed in time-based environment as a Time-Domain VF (TDVF) method for macromodeling the multiport linear system and supported with accurate validations, as described in [20]. This research proposes not only different approach towards the macromodeling of experimentally-based data system in time-domain, but also generalize the VF algorithm to be applied for all power curves and measured data within any domain.

The main aim of the proposed modifications to the VF is to generalize the VF algorithm from the s-domain (frequency environment) to the time-domain, or even for any domain application. Thus, the RAF must be converted back into a time-based formula. Therefore, the first step of this stage is to change the state space of the VF output into an RAF form, as in (8), followed by the second step, which is to substitute *jw* for each s (*h*(*s*) = *h*(*jw*)). The final step is to retrieve the originality of the input or measured data points of the modeled system; this step depends on the preliminary assumption that was adopted in (1) to describe the measured data. Thus, the resultant modeling equations can be expressed in two forms: magnitude and/or phase. In the final steps of Appendix A, the software shows this stage details and the resultant modeling formula of an empirical data in terms of magnitude representation.

### Magnitude model

If the preliminary assumption of the measured data points in (1) is magnitude data in polar form, the BEVF output result is in a time-domain based modeling equation, as in the following form (12):-

Magnitudeofh(s)=Magnitudeofh(jw)=|h(jw)| (12)

For; $h_{n}(jw)=\frac{r_{n}}{jw-p_{n}}$; for *r*_*n*_, and *p*_*n*_, real numbers, at n = 1; then:

$$\left|h_{1}(jw)|=(r_{1}/\sqrt{w^{2}+{p_{1}}^{2}}\right)$$ (13)

In general, substitute (13) and (12) in (8) for |h(t)|, the result would be as follows:-

$$h(t)=\left(\frac{\sqrt{{im(r_{1})}^{2}+{re(r_{1})}^{2}}}{\sqrt{{\left(t-im(p_{1})\right)}^{2}+{re(p_{1})}^{2}}}\right)+\left(\frac{\sqrt{{im(r_{2})}^{2}+{re(r_{2})}^{2}}}{\sqrt{{\left(t-im(p_{2})\right)}^{2}+{re(p_{2})}^{2}}}\right)+\cdots \;\ldots +\left(\frac{\sqrt{{im(r_{n})}^{2}+{re(r_{n})}^{2}}}{\sqrt{{\left(t-im(p_{n})\right)}^{2}+{re(p_{n})}^{2}}}\right)+|K|$$ (14)

Thus, in more general h(t), can be in a form (15):

$$h(t)=\sum_{c=1}^{n}\left(\frac{\left|r_{c}\right|}{\sqrt{{\left(t-im(p_{c})\right)}^{2}+{re(p_{c})}^{2}}}\right)+|K|$$ (15)

Where the index c represents the counter of the equation fractions counts till reach the index n which denotes the modeling order.

### Phase model

If the preliminary assumption regarding the measured data points in (1) is that phase data are in polar form, the BEVF output result is a time domain-based modeling equation, as in the following form (16):

Phaseofh(s)=Phaseofh(jw)=∅(h(jw)) (16)

For example, for n = 1 in (16),

$$\emptyset (h_{1}(jw))=\tan^{-1}\;\left(\frac{im(r_{1})}{re(r_{1})}\right)-\tan^{-1}\;\left(\frac{w-im(p_{1})}{re(p_{1})}\right)$$ (17)

In general, substituting (17) and (16) into (8) for ∅(h(t)) results in the following:

$$h(t)={tan}^{-1}\left(\frac{im(r_{1})}{re(r_{1})}\right)-{tan}^{-1}\left(\frac{t-im(p_{1})}{-re(p_{1})}\right)+{tan}^{-1}\left(\frac{im(r_{2})}{re(r_{2})}\right)-{tan}^{-1}\left(\frac{t-im(p_{2})}{-re(p_{2})}\right)+\cdots \;\ldots \;\ldots +{tan}^{-1}\left(\frac{im(r_{n})}{re(r_{n})}\right)-{tan}^{-1}\left(\frac{t-im(p_{n})}{-re(p_{n})}\right)+{tan}^{-1}(K)$$ (18)

$$h(t)=\sum_{k=1}^{n}{tan}^{-1}(\frac{im(r_{k})}{re(r_{k})})-{tan}^{-1}(\frac{t-im(p_{k})}{-re(p_{k})})+{tan}^{-1}(K)$$ (19)

Thus, (15) and (19) are the mathematical modeling representation of the experimentally measured data for a particular system that is in the time domain in this analysis.

**Fig 3:**
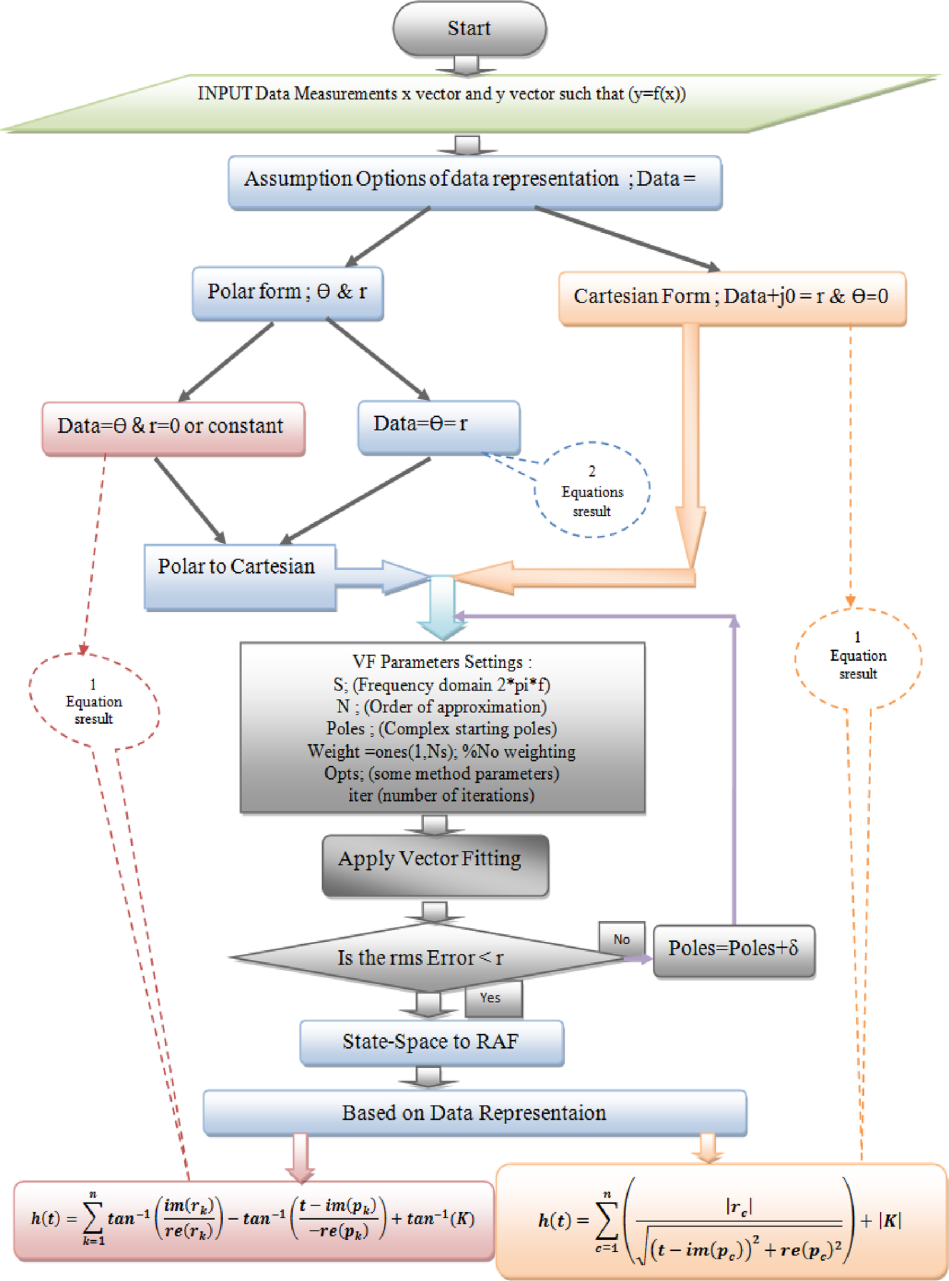
Flowchart of the proposed algorithm.

Therefore, the proposed BEVF algorithm provides the coefficient values (r_n_, p_n_, and K) for Eqs (15) and (19), where n denotes the order of the proposed modeling equations and the associated system. The designed method also provides a comparison with the standard modeling equations and shows the fitting convergence of the process. The flow chart of the proposed algorithm can be described as shown in Fig 3.

## Case studies

This paper presents the modeling of the four features of power profiles in green energy applications: two in wind energy applications, one in solar power applications, and one regarding the load demand power curve. These cases were selected based on the availability of data and previous related studies.

### Wind power profile modeling

#### Overview

A wind turbine generator transforms wind power into electric power. Its output power varies over a wide range from zero to the full capacity value based on the wind speed variation. The varying power generation and the uncertainty related to the power level result in considerable challenges in the planning and management of a power system to meet the demand-accepted level of power reliability. One of the most important tools in the wind energy industry is the accurate modeling of wind turbine power curves that significantly aid in the evaluation and monitoring of turbine power system performance, forecasting, and sizing the capacity of the wind energy storage.

The power of air movement of a mass of air flowing through a particular area *A* with a speed of *v* at a specific time t is given by:

$$P(t)=\frac{1}{2}\rho Av{\left(t\right)}^{3}$$ (20)

where *ρ* is the air density and is approximately 1.22 kg/m^3^ and the energy in kWh is:

$$E=P(t)T=\frac{1}{2}\rho A\Delta t\sum_{i=1}^{N}v_{i}^{3}$$

Therefore, when the fluctuations of the wind are taken into account, the energy obtained from a flow of air over a period of time (*t*) is given by the sum of the overall wind speeds at proportional time intervals. Usually, the hourly speeds are measured as an average (24 time buckets per day).

Since the power of the wind is proportional to the cubic wind speed according to (20), it is important to have detailed information regarding the geographic site’s wind characteristics because small errors in wind speed prediction can have large effects on the aggregated energy.

#### Wind power related studies

Several studies have evaluated statistical algorithms to fit the wind power empirical curve [21]. A few previous related studies classify the methods into parametric and non-parametric algorithms to model the wind turbine generator [21–23]. Non-parametric techniques do not impose a particular model and can be adapted to produce an estimate of the power curve, which should be fitted to the observed data. Such techniques have an important advantage over parametric techniques because they can accurately model a wide range of shapes of power curves of the system. The use of a neural network is considered a non-parametric technique utilized in back propagation and feed-forward multilayer perceptron as a generalized mapping regression procedure [24, 25] while the fuzzy logic method, as described in [25], is used to forecast the output power of a wind turbine generator as a fuzzy cluster model.

The use of the polynomial regression method to model and fit the power curve is well known. This technique has been attempted extensively in previous studies, but suffers from its global nature and sensitivity to anomalies in observations. Therefore, a high degree polynomial regression model is required to provide a good fit to the measured data points [26], but the obtained power curve of this technique may closely track the noise of the measured power data points. Therefore, a locally weighted polynomial regression, presented by [27], is used as a non-parametric method to avoid such problems. Other research have studied wind power curve fitting [28, 29] and considered the Cubic Spline Regression as another non-parametric technique. However, issues include choosing the number and location of what is called knots to fit the cubic spline model. Furthermore, though these models have good performance for fitting the wind turbine power curves that are smooth, this performance could be undesirable when the knots fall outside the boundary. In [30], the author proposed modifications to the natural cubic spline to improve the fit of the cubic spline regression model, which has been similarly addressed by [19,30–33].

Most of the methods mentioned above have good flexibility and are simple to implement and less sensitive to observable anomalies, but they either are not clear about the modeling equations, such as those of non-parametric techniques, or have insufficient modeling accuracy, especially when the observation data have sharp fluctuations. The proposed technique provides a robust method to extract the modeling equations and smooth data. The proposed Bode equation vector fitting (BEVF) algorithm may also be used to obtain the characteristic power curve of the wind turbine generator for power forecasting and real-time monitoring comparison.

In this research, the main objective is to model the wind turbine power curve with the different modeling equations listed in Table 1 and then extract the proposed modeling equations of BEVF as a new alternative modeling method to validate the obtained results and compare the RMS error values.

#### Wind power modeling

This section discusses, analyzes and evaluates the most current fitting and modeling methods available and compares them with the proposed modeling technique, which is implemented on two sub-case studies: the first one is the wind turbine FL-255 power curve, and the other is the wind speed pattern.

#### Case one: Wind turbine FL-255 power curve

**Fig 4:**
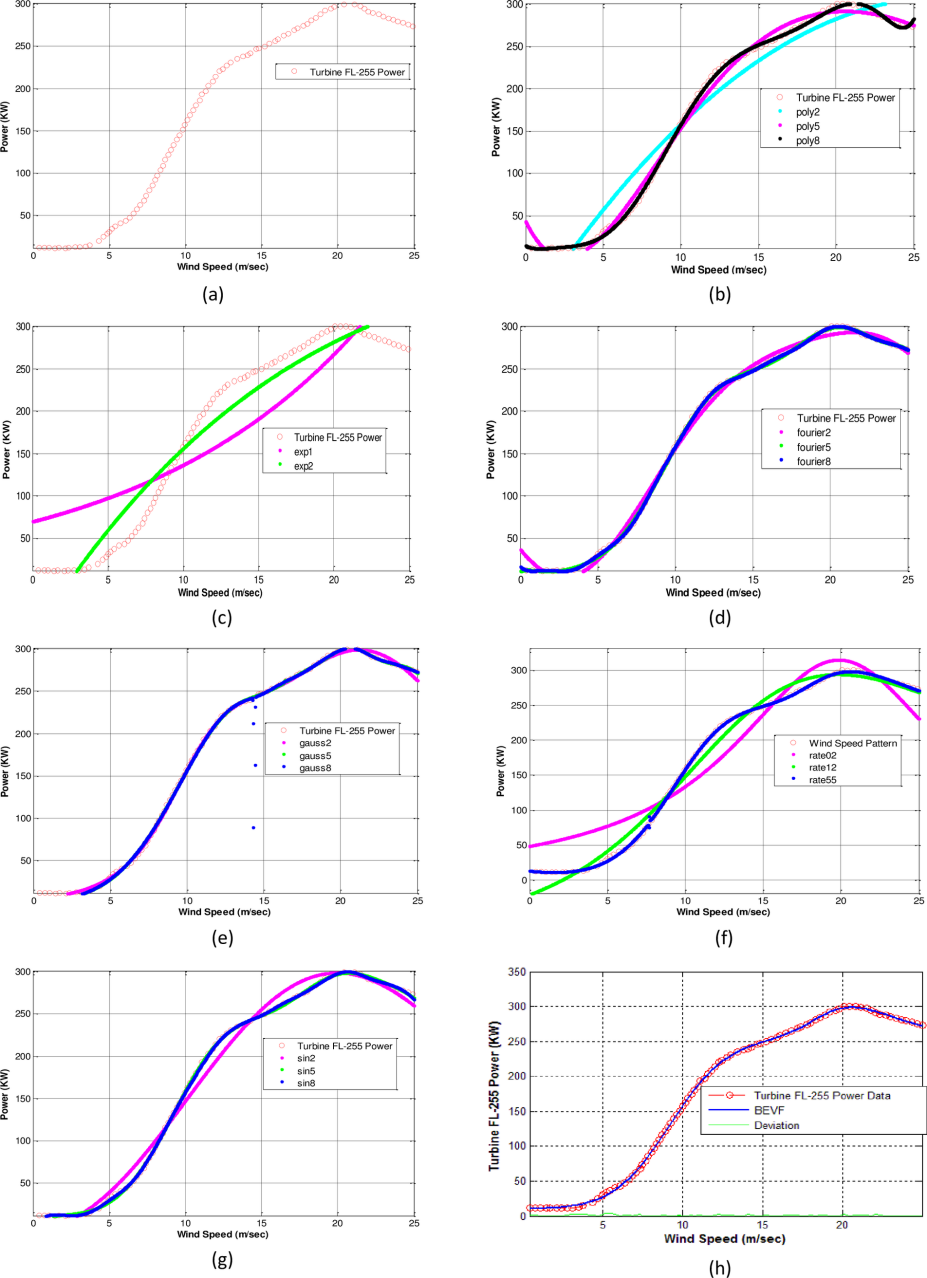
The modeling of FL-255. (a) Manufacturer’s power curve. (b) Polynomial of degree 2–8 models with RMS error = 2.8463. (c) Exponential of degrees 1 and 2 and RMS error = 26.1715. (d) Fourier series of degrees 2–8 and RMS error = 0.85997. (e) Gaussian of degrees 1–3 and RMS error = 3.1356. (f) Rational with degrees 02, 12 and 55 and best RMS error = 1.6787. (g) Sum of sine of degrees 2–8 and best RMS error = 1.6654. (h) Proposed (BEVF) modeling of 8 degrees and RMS error = 0.66706. The red circle curves represent the theoretical power curves from the manufacturer.

The wind speed versus the output power data set (v_k,p_k) is plotted from the manufacturer’s power curve. The wind turbine model FL-255, manufactured by Vestas Wind System A/S with a power of 250 kW, is considered to present the power curve. Fig 4(A) represents the theoretical power curves for the turbine (see S1 File).

Fig 4 shows the modeling of the turbine FL-255 power data with the different parametric modeling equations. The detailed results of the fitting process in this case study, which include the model equations, associated parameter values, and the RMS error, are listed in S1 Table.

#### Case two: Wind speed pattern

**Fig 5:**
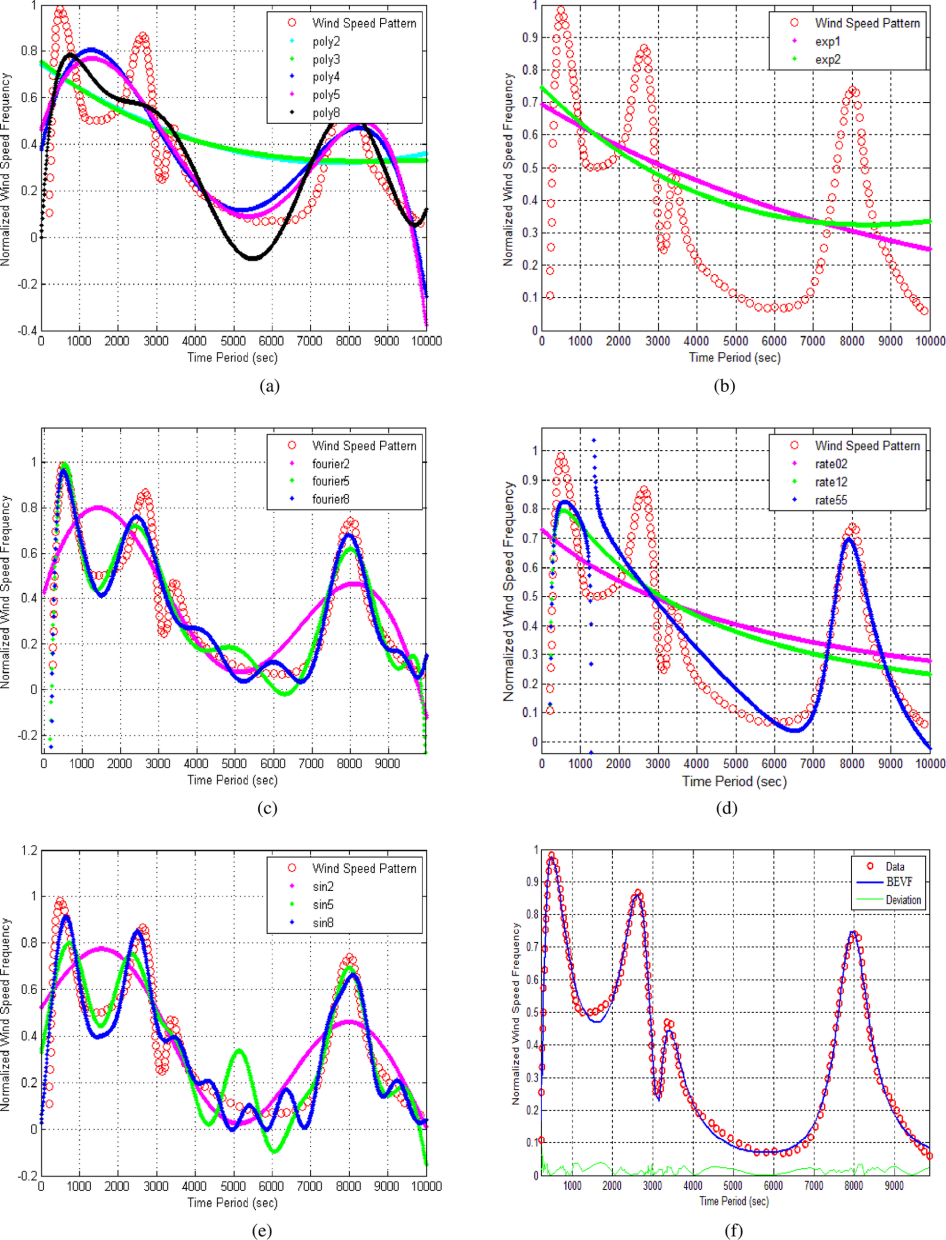
The modeling of the wind speed pattern. (a) Polynomial of degree 2–8 regression models and best RMS error = 0.1468. (b) Exponential with degrees 1 and 2 and best RMS error = 0.2162. (c) Fourier series from degrees 2, 5 and 8 and best RMS error = 0.0709. (.) Gaussian not applicable. (d) Rational with degrees 02, 12 and 55 and best RMS error = 0.11947. (e) Sum of sine with degrees 1–3 and best RMS error = 0.0889. (f) Proposed (BEVF) modeling with 16 degrees and RMS error = 0.0204. The red circle curves represent the theoretical wind speed pattern.

The wind speed pattern may be represented as a spectrum of wind speeds. Higher values of this pattern indicate a considerable variation in wind speed over the equivalent time interval [34]. Such patterns are important for wind power aggregation estimations and forecasting. Fig 5 shows the application of various parametric modeling equations for this case study as well as for the implementation of the proposed BEVF modeling (see S2 File).

### Load power curve estimation

#### Overview

From previous studies, several accurate models describing the time-varying behavior was investigated in both physical and empirical systems. However, a more general mathematical modeling algorithm to interpret most physical measurements of power systems is still required. There is also a need to accurately model the collective load at different power collection points in the generation and consumption network [35]. It is possible to determine the model of a particular load and bring it to higher levels, but as the number of appliances increases or perhaps only the variety of individual consumption increases, the complexity of the model also increases [36]. Therefore, the proposed method is considered an efficient alternative modeling method for this application.

The data of a load power are crucial for managing electricity generation and consumption within the network to predict the optimal production capacity. Therefore, an accurate understanding of the appliance power is important, especially when small scale energy is optimally sized in terms of the network or demand. This understanding is also useful for managing medium scale networks for residential applications. The measured data from the electrical network of the utility for the domestic electricity consumption do not include sufficient information about its characteristics. The power curve data are usually aggregated appliance consumptions without information about the occurrences in the individual appliances. Therefore, the fluctuation in electricity consumption remains hidden, as does the distinction of consumption among different types of appliances [37–39].

P. Price [40] offered a few suggestions about the graphical display of the power data to describe the load power shape and introduced some techniques to describe the shape of the power load statistically based on linear regression modeling of real data to adjust for the weather effects. The load data profiles that contain details regarding the consumption can be created with bottom-up load models [41]; here, the load is constructed from elementary power components that could be a load system or individual appliances. In [41], a few to up to a few thousands of appliances were examined on an hourly basis. Finally, actual wind and load power estimated data over a period of 24 hours were studied in [42].

#### Electric load shape and its variability

In this case study, we apply the proposed modeling method to electric load curve data and the variation of power consumption over a daily time period with a sampling rate of 15 min/sample to improve our understanding of power load variable characterization and the correlations between the source power and load.

**Fig 6:**
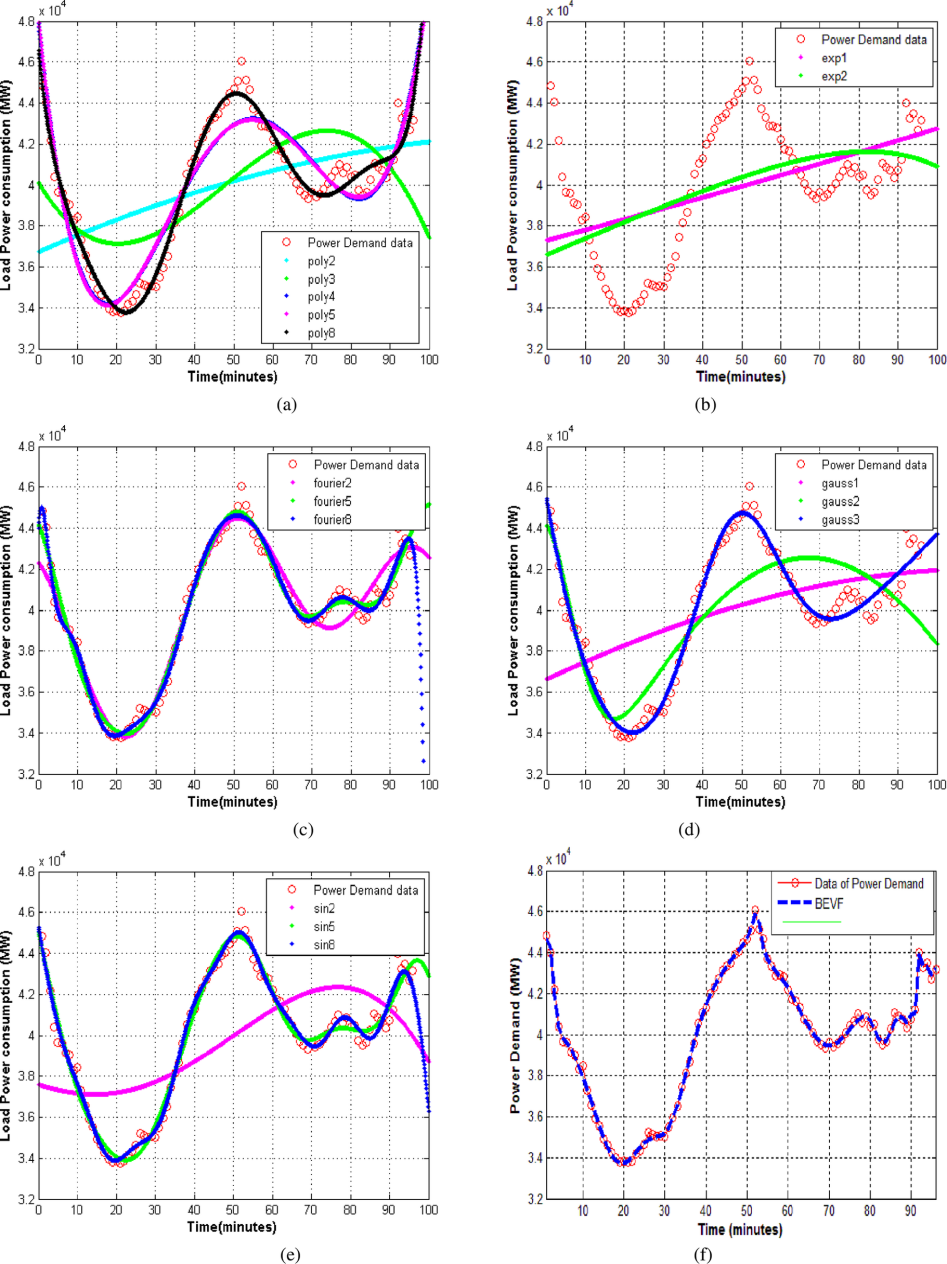
The modeling of the power observation data from the RTE information system that shows the variations in quarter hourly points of French power consumption during the day of 20/8/2016. (a) Polynomial of degree 2–8 regression models and best RMS error = 627.9829. (b) Exponential with degrees 1 and 2 and best RMS error = 2.9238e+03. (c) Fourier series of degrees 2–8 and best RMS error = 434.4140. (d) Gaussian and best RMS error = 598.6974. (.) Rational with degrees 02 and 12 not applicable. (e) Sum of sine with degrees 2–8 and best RMS error = 462.3307. (f) Proposed (BEVF) modeling with 30 degrees and RMS error = 191.4442. The red circle curves represent power observation data.

The objective of this research is to expand on an alternative-based modeling approach that has the ability to predict the power consumption of aggregated appliances and solve the complexity problem of sharp variations in load power curves. As a case study in this branch of applications, we used the online power data of real-time measurements of production from France’s National Control Center (CNES); the information provides load curve data points and the forecast generated every day [43], (see S3 File), as shown in Fig 6.

### PV power forecasting

#### Overview

**Fig 7:**
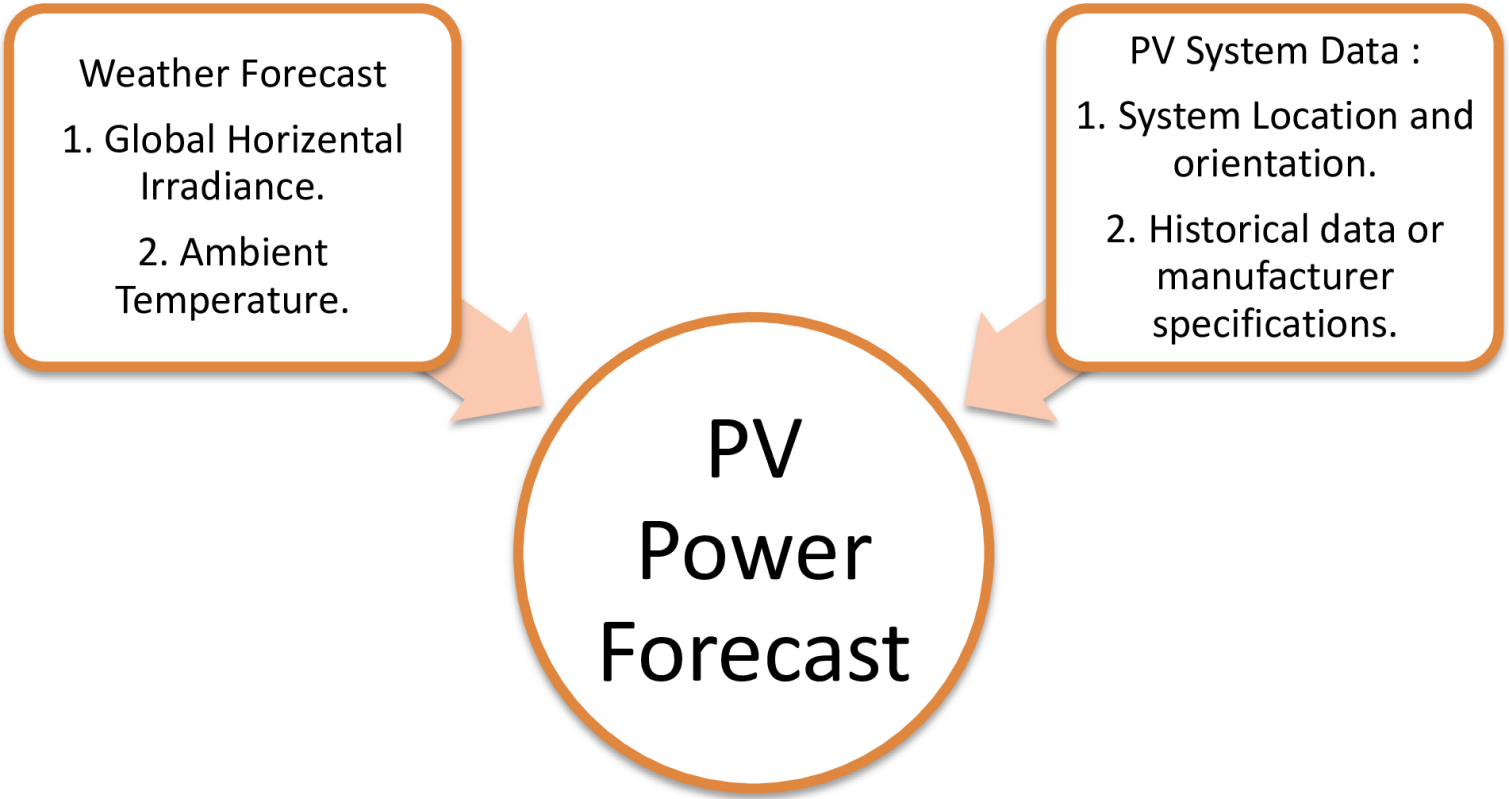
The typical approach for generating a PV power forecast.

PV systems are highly influenced by the two main challenges of their penetration rates: uncertainty and variability. Therefore, a PV output reveals the variability over the year, which is difficult to estimate, similarly for the uncertainty. The method used to address these challenges is PV forecasting. The fundamental concept of this typical approach is shown in Fig 7.

The major variables controlling the output PV power are the irradiance and the ambient temperature of PV modules. Other variables, such as the incidence angle and the spectral distribution of radiance, are integrated for some PV modules. Based on data availability, the PV modules may either be fitted via historical data, as described in [44], or may be based on the manufacturer’s specifications, as in [45, 46].

Referring to Fig 1, the two major types of modeling that can be utilized to estimate the output power from given inputs are as follows:

1. Parametric modeling, which considers the PV as a known box and uses several parameters to construct the model.
2. Non-parametric modeling, which considers the PV as a black box and does not include any information about the properties of the system. However, this type of modeling is based mainly on the data to estimate the performance of the system from historical statistical data over time for a series of inputs/outputs. Examples of this type of modeling include artificial neural networks (ANN), data mining, the adaptive network-based fuzzy inference system (ANFIS) mode, clustering methods, wavelet support vector machines, and the joint probability of wind speed and power.

The two approaches could use the same effective inputs, such as irradiation and temperature, based on the same output.

Several studies addressed the forecasting of the PV power. The mismatch power losses in PV systems are mainly caused by the partial shading of solar arrays but can be reduced by replacing the module connections of a PV array [47]. The field data measurements are utilized to identify module parameters individually and for all system elements [48]. Short term hourly solar PV forecasts for horizons between 0 and 48 h ahead based on the Global Environmental Multiscale model are studied over a one-year period to train the forecast of the PV solar array as in [48,49].

Since the PV array output power depends on solar irradiation and ambient temperature, the full use of this power is achieved when the Maximum Power Point Tracking (MPPT) is adopted. One of the techniques employed to use this process is the Perturb and Observe method, which is simple in its control structure but suffers from the steady-state oscillations that appear from the perturbation. Thus, one of the solutions is to build a forecasting model of MPPT supported by the Perturb and Observe method [50].

Regression modeling is used in a PV array condition monitoring system in an on-line parameterization process that is based on the PV array irradiance and module temperature. By using the predicted and measured PV array output power values, this monitoring system is able to detect power losses of 5% in the PV array [51].

One of the motivations for this research originated when our previous study exposed difficulties with the solar power profile in PV-powered home systems through the design of a new charge controller, as mentioned in [52], and with household power consumption curves when a DC voltage matching concept was used, highlighting a need for modeling the power system curves [53].

Thus, to address the cyclic and unpredictable variations of PV array output power, the BEVF algorithm is used in this paper as a solution to extract the modeling equations that govern these variations to achieve good fitting convergence and forecasting results.

#### Forecasting photovoltaic array power and modeling

**Fig 8:**
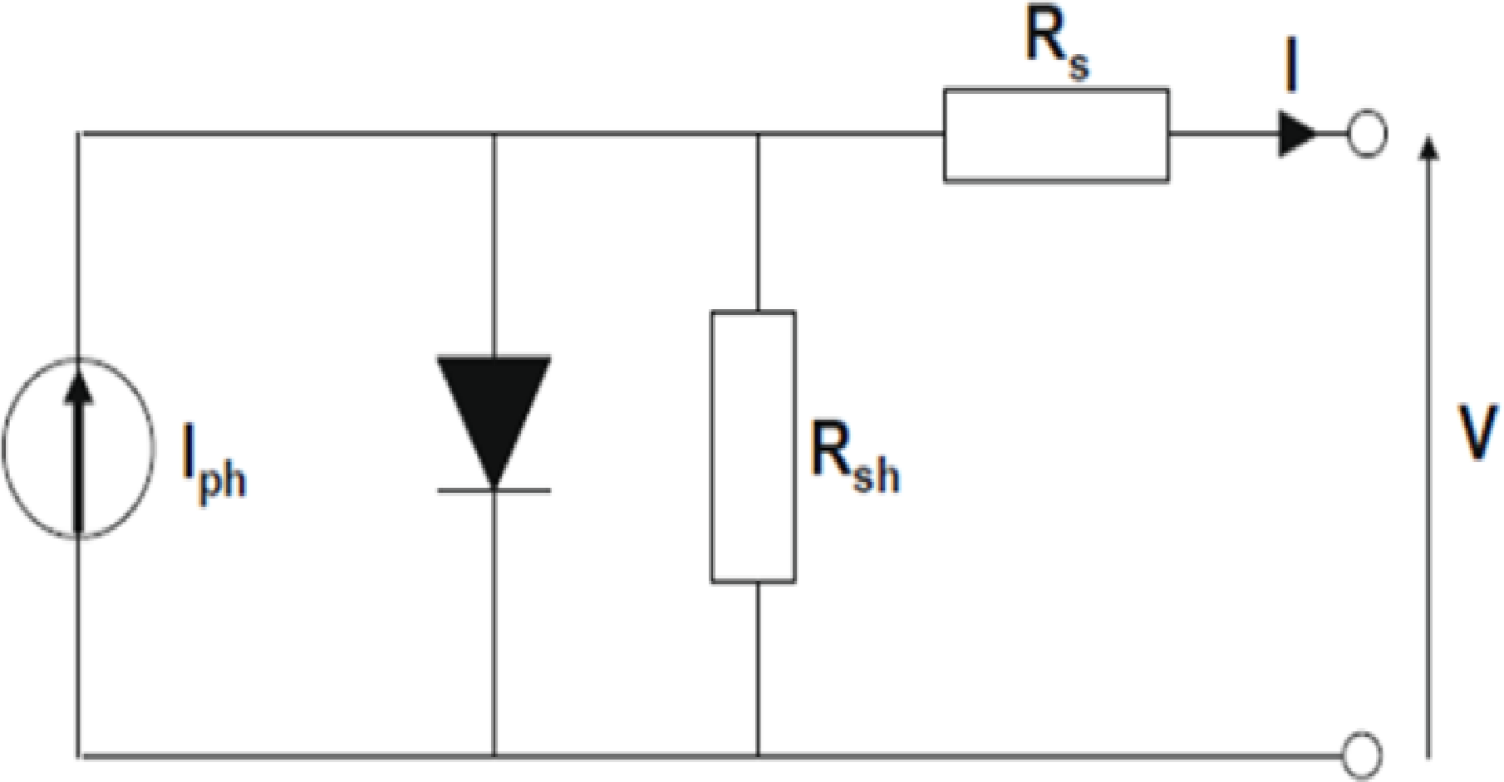
Equivalent circuit of photovoltaic module.

For solar cells, a single-diode model is commonly used to model the crystalline silicon solar module. Many studies have described the modeling of the solar cell, as mentioned in the literature above, and the single-diode equivalent circuit is shown in Fig 8.

In this paper, we discuss the generation power curve modeling for monitoring and forecasting purposes. Many factors significantly affect the power curve shape in solar power generation systems such as the fluctuation of light shading and the ambient temperature changes.

This research evaluates the modeling of aggregate forecasts of PV solar power generation over several days. The data of the solar observations have been collected from an official on-line website belonging to the Elia Group, which is Belgium’s high-voltage transmission system operator (30 kV to 380 kV) [54]. This website offers published forecasts that are day-ahead forecasts up until three days into the future and one intraday forecast, updated once a day at 11 am. The latest information for both types of forecasts are obtainable through the website’s data-export functionality.

**Fig 9:**
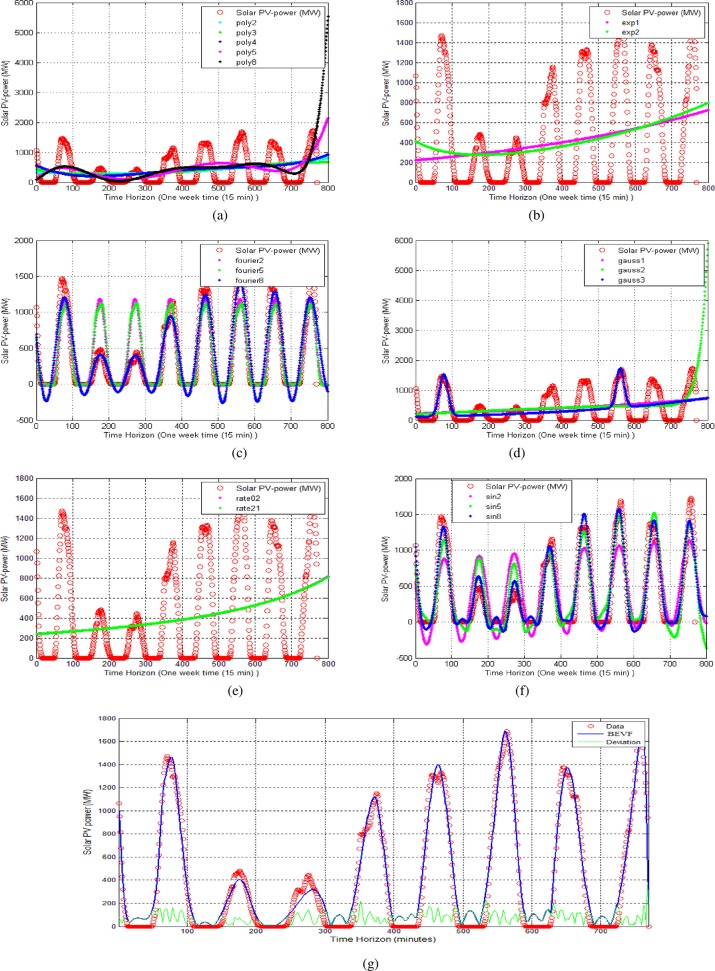
The modeling of seven-day solar output power. (a) Polynomial of degree 2–8 models and best RMS error = 458.2729. (b) Exponential of degrees 1 and 2 and best RMS error = 493.3065. (c) Fourier series of degrees 2, 5 and 8 and best RMS error = 174.5701. (d) Gaussian and best RMS error = 402.4050. (e) Rational of degrees 02 and 12 and best RMS error = 495.0226. (f) Sum of sine of degrees 2–8 and best RMS error = 123.2312. (g) Proposed (BEVF) modeling with 40 degrees and RMS error = 88.5866. The red circle curves represent the theoretical seven-day solar output power.

The solar observations versus the power output data set are plotted from the on-line Elia website and represent the long term solar power curve. Fig 9 shows the application of the different parametric modeling equations in the case of one week and 15 minutes per sample [54], (see S4 File).

## Fitting results evaluation

Several metrics for statistical data can be considered to appropriately measure the model performance in the fitting of power observations such as the Root Mean Squared Error (RMS error). In this research, we use the RMSE formula as in (21):

$$RMS\;error=\sqrt{\frac{1}{n}\sum_{i=1}^{n}{\left(p_{i}-p_{ei}\right)}^{2}}$$ (21)

where p_i_ is the observed power data and p_ei_ is the estimated value of this power.

**Fig 10:**
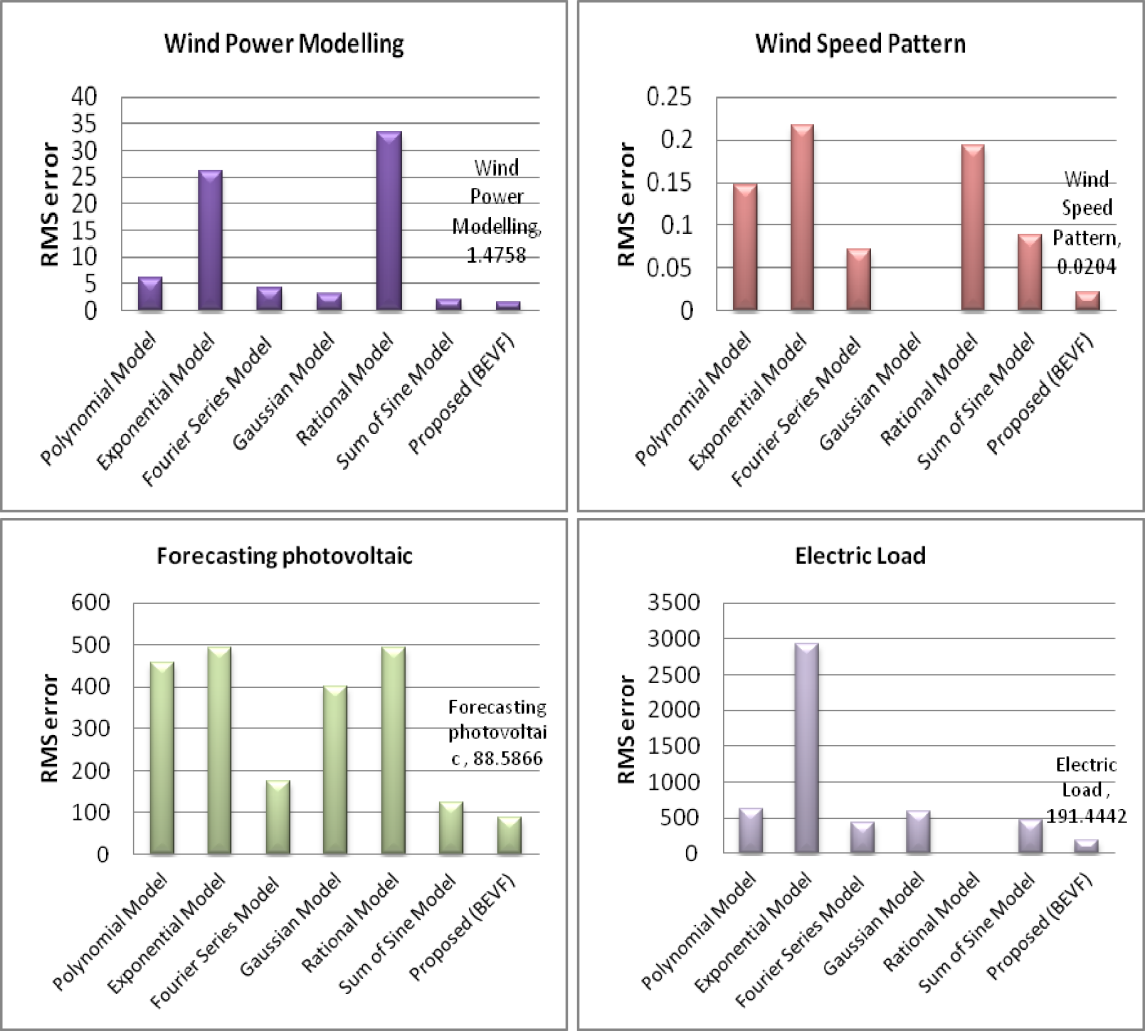
RMS error bar chart for the four case studies: Wind turbine FL-255 power curve, wind speed pattern, forecasting photovoltaic array power, and electric load demand.

Regarding the performance of the error analysis, the values of the RMS error of the polynomial regression (Poly), exponential regression (exp), Fourier series (Fourier), Gaussian (gauss), rational (rate), sum of sine (sin), and proposed (BEVF) methods are analyzed here, as shown in Fig 10.

In this paper, we present a mathematical modeling approach to enhance the understanding of mathematical modeling of measured or calculated data and to model future energy sources and load consumption profiles.

**Fig 11:**
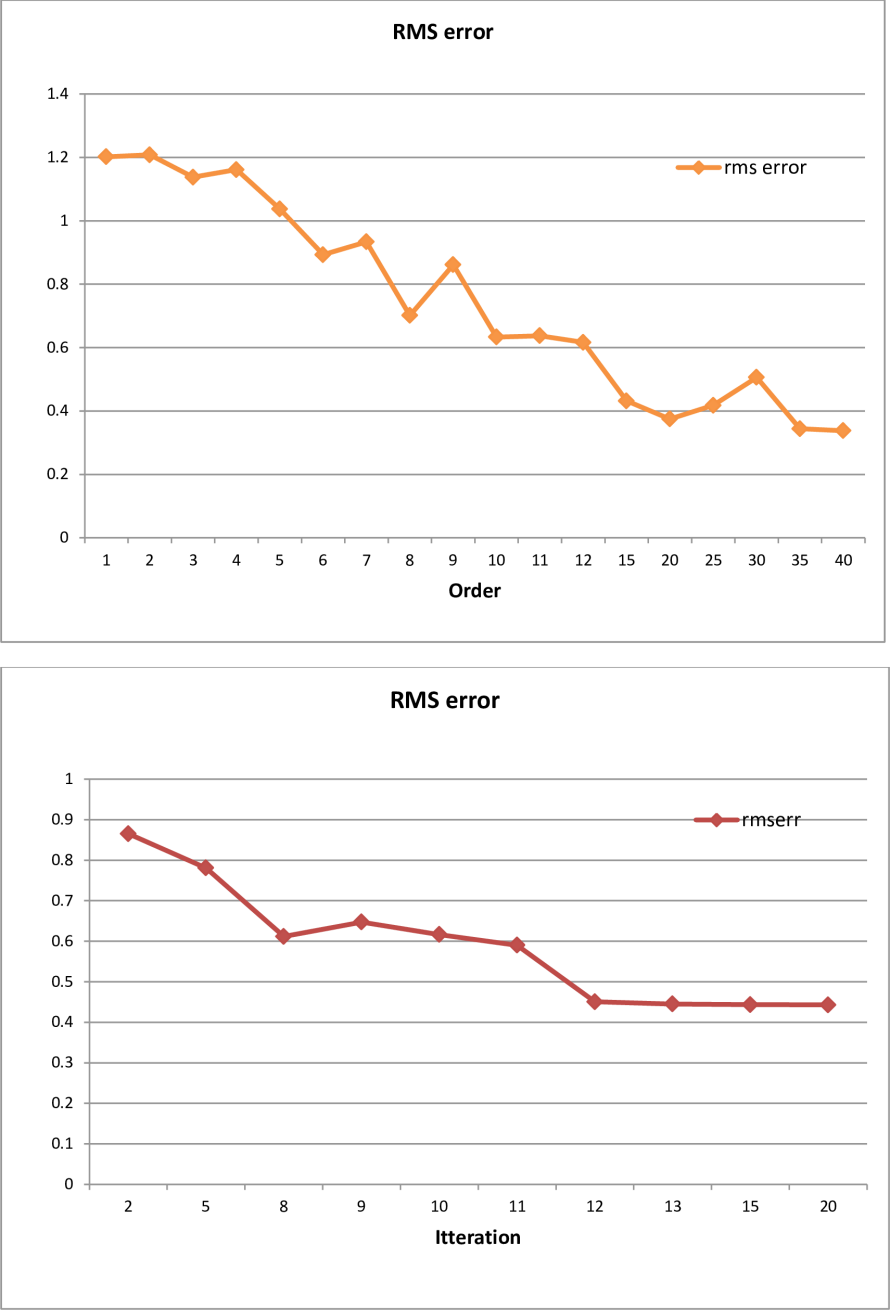
RMS error versus order (left), and versus iteration (right).

For the proposed BEVF algorithm, the order effect on the results accuracy of the modeling equations has been investigated and presented, as shown in the left of Fig 11, while the effect of the iteration number on the results is presented in the right of Fig 11. It is evident that the adequacy and accuracy of the modeling improve with the rise in the resultant order and iteration, but consequently, higher order implies higher complexity of the mathematical equation.

**Fig 12:**
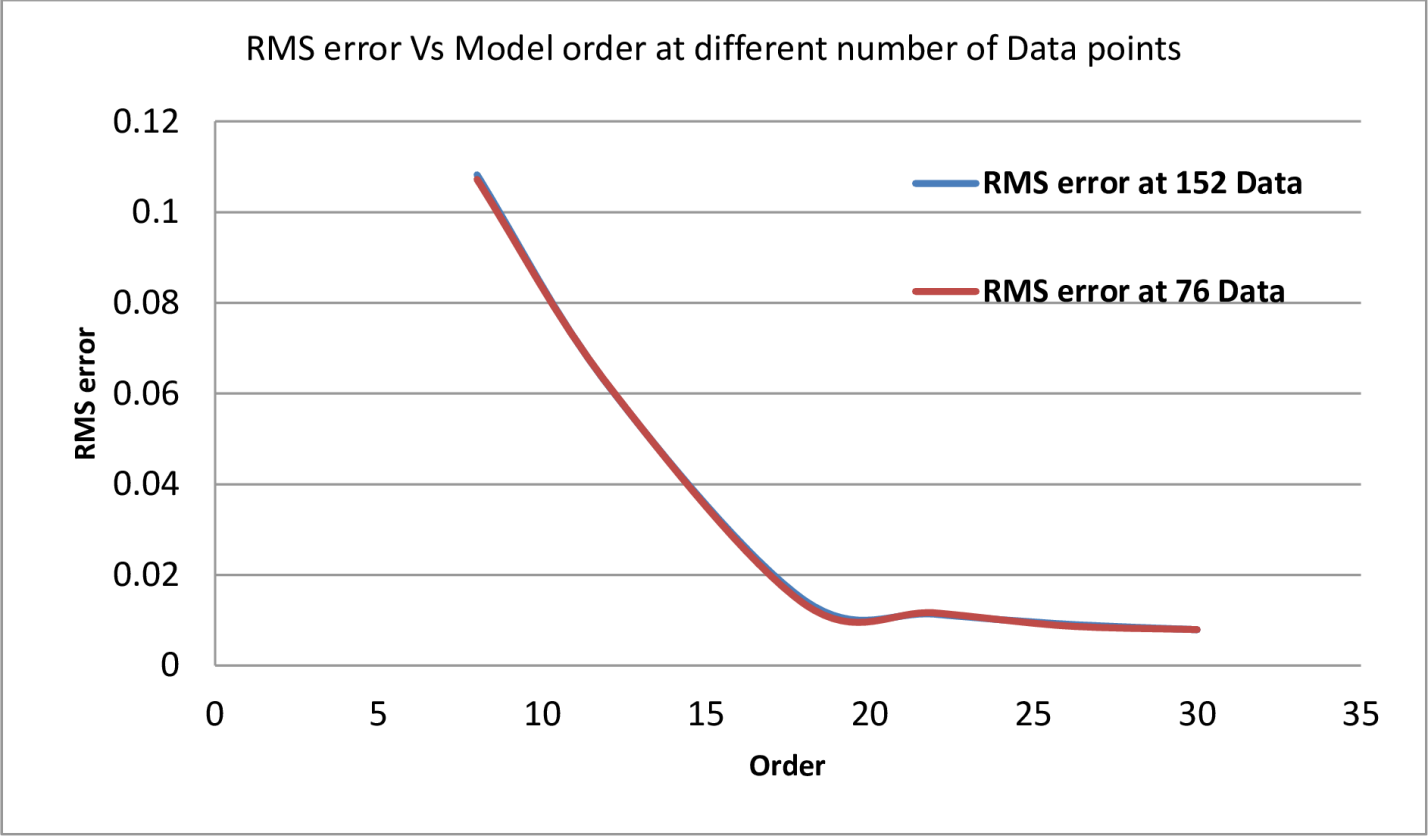
The effect of the number of sampling data set on the modeling performance.

To clarify the effect of the number of sampling data on the modeling, we considered the data of Case Two (Wind Speed Pattern), the comparison performed under two sets of data, 152 and 152/2 samples. A relation between the RMS error and the modeling equation order, for the two data sets, is clearly shown in Fig 12.

## Conclusions

The complexity of a particular model involves a trade-off between simplicity and accuracy. This paper proposes a new method with a significant modification to the vector fitting method. The modification generalizes the application of the VF method to the modeling of any measured data or field observations, as discussed in the four case studies. Furthermore, this method can model a wide range of field data in the form of a dependent function f(x), whether x represents time or another independent variable.

Modeling complexity arises when the measured data are represented by sharp changes or rapid fluctuations. The method of setting the initial poles and distribution over the x-axis directly affects the results. The final mathematical equation for the power generation data is strongly affected by the power profile shape, which provides an indication for parameter settings for the algorithm and, hence, the complexity or the order of the modeling equation.

In the wind energy field, the performance of the proposed method is evaluated based on two common wind power analyses using data modeling: wind turbine generator modeling and wind speed pattern data sets. A wind turbine (FL-255) power curve is selected for the analysis of the performance of the presented methods to be compared with our proposed BEVF method. The fit accuracy of each modeling method is evaluated using the RMSE metric. The result of this analysis can be used in different applications such as wind turbine performance monitoring, real-time power forecasting, and size estimation for the energy storage capacity of wind power system integration.

In forecasting photovoltaic array power, the performance of the PV power system is mainly influenced by the site’s weather, irradiance and temperature. This study also presents the modeling of a photovoltaic power system based on the (BEVF) algorithm method and compares this modeling with the current modeling equations. More accurate results of the output power can be obtained by implementing higher orders for the modeling equations. The fitting evaluation results show that our proposed (BEVF) method is effective and feasible with regard to time compared with the best fitting results of the common modeling equations.

For the electric load demand, this method has the potential to be further adapted to a varying electricity consumption background in addition to being applicable to a wider application range without much intervention. For example, on a daily basis, the sequential dependence between the daily load power curves is modeled through the assumption that the power curves from two successive days have a linear association; therefore, the modeling of one day can estimate the next day’s power demand.

The validation process has analyzed the performance of the proposed methodology according to the accuracy of the fit and the environmental parameters of the system. The main conclusions of this analysis are as follows:

- Increasing the number of power system variables may not necessarily lead to an increase in the accuracy of the modeling or forecast.
- Less complexity implies lower order for the equations of the system model when greater similarity of measurements is available for periodic data.
- The number of the sampling data set has no significant impact on the modeling performance and order.
- The confidence period ability to include all observations is important to achieve higher modeling performance.
- The proposed algorithm ensures the provision of two mathematical equations in terms of the logarithm and inverse tangent forms.
- Although, the motivation of considering the vector fitting (VF) algorithm is due to its robust numerical fitting property as a mathematical method for sampled response-matching system identification, but, as per the reviewer mentioned, the proposed modification which represented by the two input and output adaptors can be applied to any algorithms that estimating models using frequency-domain data to provide state-space or transfer function for the model.
- This method opens the horizon to implement another mode of system modeling such as passive component’s representation for some types of load consumption profiles such as those for motor-based appliances, e.g., pumps, air conditioners, refrigerators, and freezers.

In future studies, it is intended to implement this method into two aspects; analytically, where the proposed modification which represented by the two input and output adaptors can be applied to any algorithms that estimating models using frequency-domain data to provide state-space or transfer function for the model, and experimentally, to investigate and model household appliances and other various systems in a more detailed manner as well as to utilize the same modeling results to physically simulate the system with its equivalent RLC modeling.

## Appendix A

We Consider here an example to demonstrate the algorithm steps in some details as well as to provide a proof of concept for the proposed method.

Let us take the Normal Distribution or Bell Curve, which is given by Equation (B.1)

$$y=\frac{1}{\sigma \sqrt{2\pi }}e^{\frac{{-(x-\mu )}^{2}}{{2\sigma }^{2}}}$$ (B.1)

Where μ and σ represent the mean and standard deviation respectively. The problem here is to find the alternative fitting equation that may equivalent to (B.1) by using the proposed method.

1. **Plotting (B.1) in the following commands**:
  mu = 2; % mean
  segma = 0.5; % standard Deviation
  x = 1:0.1:5; % range of data
  y = (1/(segma*sqrt(2*pi)))*exp(-(x-mu).^2/(2*segma^2));
  figure, plot(x,y)
2. **Input adaptor** **(Assumptions for VF function initialization)**
  w = x; % let the independent variable x is w
  r = y; % let r (magnitude of FR)represents the dependent variable y
  th = y; % either to let the phase (th) = y = r or th = 0
  [re,m] = pol2cart(th,r); %Transform polar to Cartesian coordinates
  f = (re+m*1i);    % the VF complex function of the input data.
  s = 2*pi*1i.*w;   %
  Ns = length(s);   % Number of data poits
  N = 6;            % order of approximation
  poles = -linspace(1,3,N); % Initial poles
  weight = ones(1,Ns); %All frequency points are given equal weight
3. **VF method**:** % provides State Space matrices**
  A, B, C, D, and E.
  Rmserr     % root mean square error
4. **Output adaptor** **(Transformation)**
  [num,den] = ss2tf(A,B,C,D,1);
  [r,p,k] = residue(num,den); % Rational Approximation function parameters
  syms t rmag
  rmag = 0;
  for kk = 1:length(r);
      if abs(real(r(kk))/imag(r(kk))) > 1e+5
          r(kk) = real(r(kk));
      end
      if abs(real(p(kk))/imag(p(kk))) > 1e+5
          p(kk) = real(p(kk));
      end
        rmag = rmag + (sqrt((real(r(kk)))^2+(imag(r(kk)))^2)/sqrt((real(p(kk)))^2+(imag(p(kk))+t)^2));
  end
  rmag = vpa(rmag)+k;
  Mag = simplify(rmag);

Where the Mag is the function that equivalent to y in (B.1), and given as a result of this algorithm as follows:

$$\begin{array}{c} Mag=\frac{2.1156}{\sqrt{\left({\left(t-10.441\right)}^{2}+14.233\right)}}+\frac{2.1156}{\sqrt{\left({\left(t+10.441\right)}^{2}+14.233\right)}}\\ \;+\frac{1.834}{\sqrt{\left({\left(t+14.21\right)}^{2}+13.132\right)}}+\frac{1.834}{\sqrt{\left({\left(t-14.21\right)}^{2}+13.132\right)}}\\ \;+\frac{3.1334}{\sqrt{\left(t^{2}+140.43\right)}}+\frac{79.924}{\sqrt{\left(t^{2}+2907.32\right)}}+1.0372 \end{array}$$

**Fig 13:**
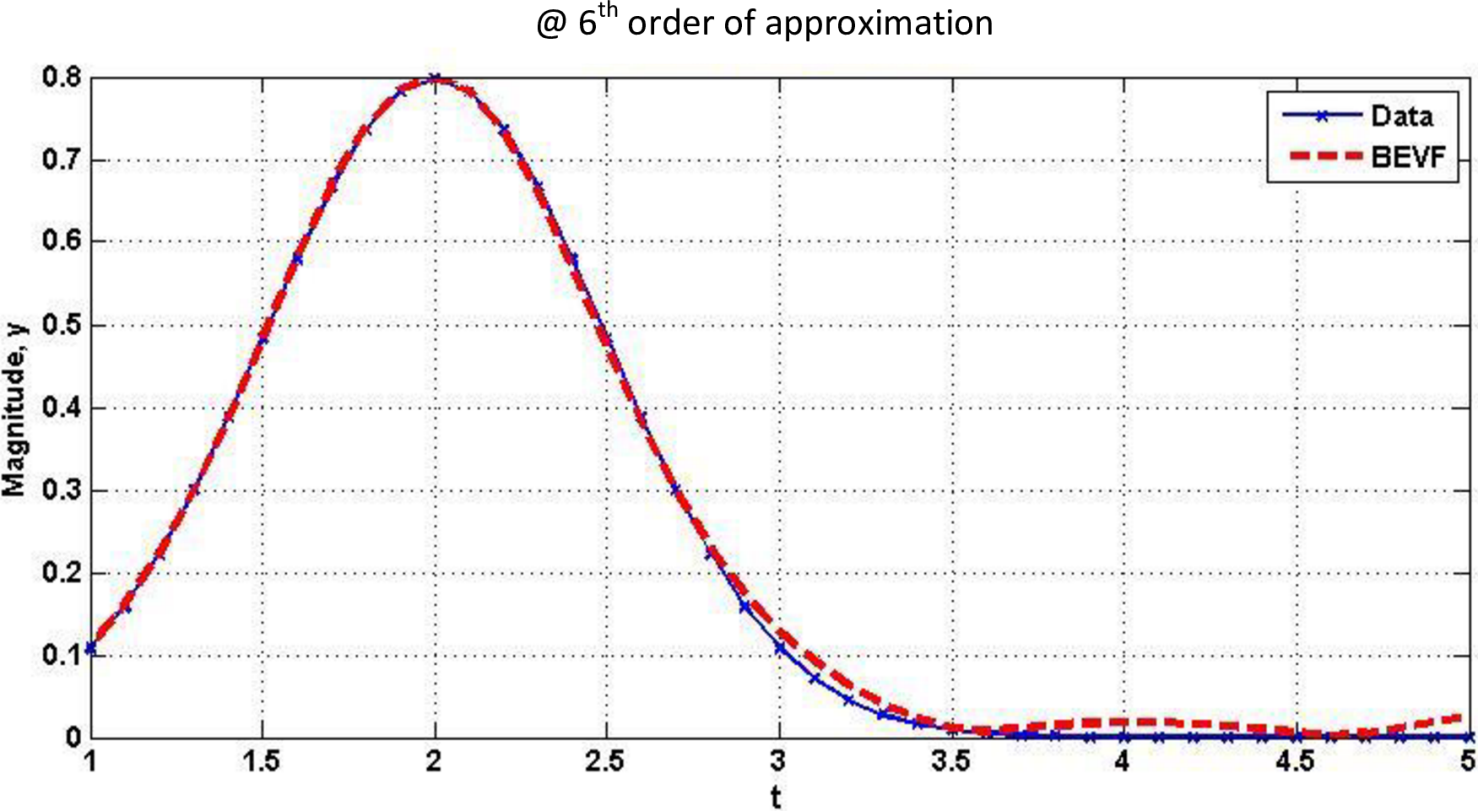
Comparative curves between the data of Normal Distribution curve and the proposed model output.

The graphical results can be seen in (Fig 13).

To evaluate and show the possibilities to model or simulate a data by the proposed method, we compared BEVF by the Autoregressive with external input (ARX) model [55][56], which also reveals a wide range of applicability for identifying the model parameters.

**Fig 14:**
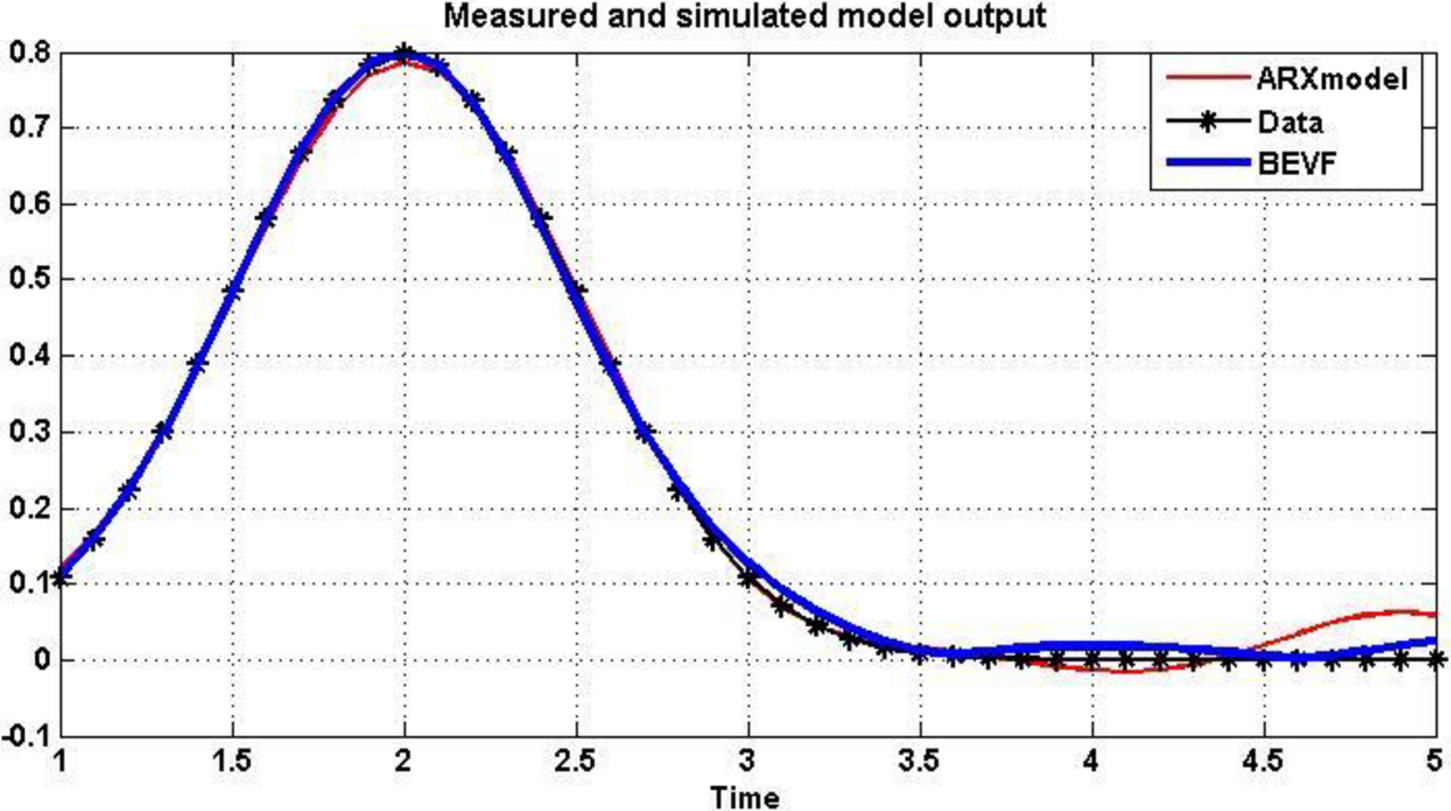
Comparison between data of (B.1), ARX and BEVF models.

Fig 14, displays the differences among the empirical data of (B.1), from one side, and the ARX and BEVF from the other side.

By using system identification functions in MATLAB, the resultant discrete-time ARX model can be described by the equation:

ARXmod=A(z)y(t)=B(z)u(t)+e(t)where

$$A(z)=1-3.632z^{-1}+5.136z^{-2}-3.346z^{-3}+0.8482z^{-4}$$

$$B(z)=0.01435-0.01536z^{-3}$$

At a sample time = 0.1 seconds.

Where the RMS error = 0.0126 for the modeling by the proposed 6th order BEVF, while it is = 0.721 when ARX model is used.

## Supporting information

S1 File MATLAB data for FL-255 turbine (Manufacturer’s power curve). (MAT)

S2 File MATLAB data of a wind speed pattern [34]. (MAT)

S3 File Excel data of an electrical load curve data and the forecast generated every day [43]. (XLSX)

S4 File Excel data for seven-days of a solar output power [54]. (XLS)

S1 Table Comparison results between the several common modeling equations and the proposed (BEVF) method for the 250 KW wind turbine model FL-255. (DOCX)
