# Supplementary material for: Field data-based mathematical modeling by Bode equations and vector fitting algorithm for renewable energy applications
Source: PLoS One. 2018 Jan 19;13(1):e0191478. doi: 10.1371/journal.pone.0191478 (PMC5774780; doi:10.1371/journal.pone.0191478)

**S1 Table.** Comparison results between the several common modeling equations and the proposed (BEVF) method for the 250 KW wind turbine model FL-255.


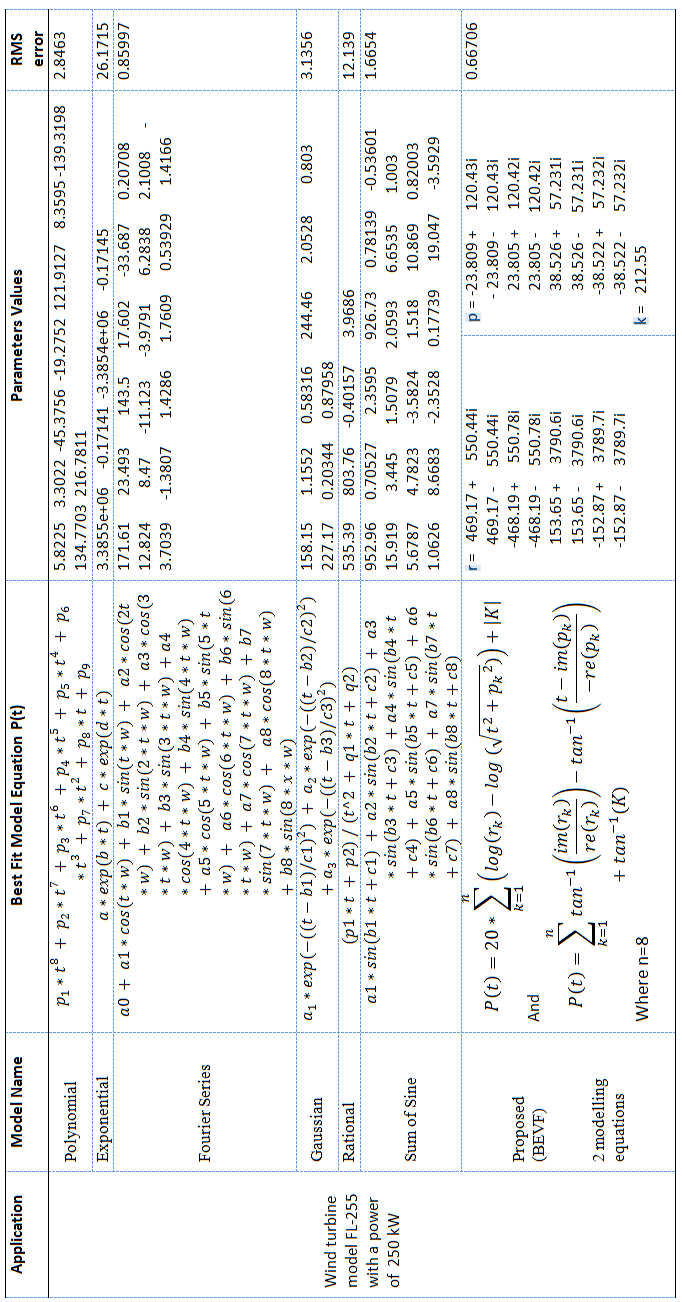

Supplement: S1 Table — (DOCX) [file pone.0191478.s005.docx]
